# The Society of Radiographers

**Published:** 1921-02-05

**Authors:** 


### The Society of Radiographers.

With a view to securing recognition of a definite professional status for qualified non-medical workers who are responsible for carrying out treatment in radiotherapy under the direction of medical officers, for taking skiagams, and for the general care and supervision of the apparatus, these workers, known as radiographers, have formed an Association under the title of "The Society of Radiographers." Its main objects are to secure adequate professional training for radiographers and to ensure that intending members have received such training and passed such examinations as may be approved from time to time. The reading and discussion of papers are also placed in the forefront of the programme, and it is hoped that at no distant date branches may be formed in various provincial centres.

The executive body consists of: President, Sir Archibald D. Reid, K.B.E., C.M.G.; Vice-Presidents, R. Knox, M.D., C. H. Wordingham, C.B.E., M.I.E.E.; a Council, consisting of six medical members, six electrical, and six radiographers, including Mr. A. O. Forder and Mr. G. F. Westlake, the well-known radiographers of King's College and the Cancer Hospitals respectively. The latter is acting as Hon. Secretary, and particulars regarding membership can be obtained from him at the Cancer Hospital, Fulham.

